# Pulse-Width Multiplexing *ϕ*-OTDR for Nuisance-Alarm Rate Reduction

**DOI:** 10.3390/s18103509

**Published:** 2018-10-18

**Authors:** Xiang Zhong, Xicheng Gao, Huaxia Deng, Shisong Zhao, Mengchao Ma, Jin Zhang, Jianquan Li

**Affiliations:** School of Instrument Science and Opto-Electronics Engineering, Hefei University of Technology, Hefei 230009, China; zhx0325@hfut.edu.cn (X.Z.); xicheng@mail.hfut.edu.cn (X.G.); zhaoshisong@mail.hfut.edu.cn (S.Z.); mmchao@hfut.edu.cn (M.M.); zhangjin@hfut.edu.cn (J.Z.); lijq@hfut.edu.cn (J.L.)

**Keywords:** *ϕ*-OTDR, pulse-width multiplexing, nuisance-alarm rate, multisensor data fusion

## Abstract

A pulse-width multiplexing method for reducing the nuisance-alarm rate of a phase-sensitive optical time-domain reflectometer (ϕ-OTDR) is described. In this method, light pulses of different pulse-widths are injected into the sensing fiber; the data acquired at different pulse-widths are regarded as the outputs of different sensors; and these data are then processed by a multisensor data fusion algorithm. In laboratory tests with a sensing fiber on a vibrating table, the effects of pulse-width on the signal-to-noise ratio (SNR) of the ϕ-OTDR data are observed. Furthermore, by utilizing the SNR as the feature in a feature-layer algorithm based on Dempster–Shafer evidential theory, a four-pulse-width multiplexing ϕ-OTDR system is constructed, and the nuisance-alarm rate is reduced by about 70%. These experimental results show that the proposed method has great potential for perimeter protection, since the nuisance-alarm rate is significantly reduced by using a simple configuration.

## 1. Introduction

Phase-sensitive optical time-domain reflectometry (ϕ-OTDR), a powerful tool for distributed intrusion sensing in perimeter protection, has attracted increasing interest since it was proposed in 1993 [1]. In a ϕ-OTDR system, light pulses of a fixed width are injected into a sensing fiber, and then, Rayleigh-backscattered light is acquired to distinguish the intrusion event, as well as its location. The sensing range is usually of the order of tens of kilometers, so the sensing fiber cable may pass across different terrains, such as deserts, swamps, rivers and lakes, and different parts of the cable may encounter different weather conditions, such as hurricane, rain, snow and hailstorms. In addition, polarization fading has an impact on ϕ-OTDR [2]. Because of the complications introduced by terrain, weather conditions and polarization fading, ϕ-OTDR suffers from a serious rate of nuisance alarms, which hinders its practical application.

To decrease the nuisance-alarm rate, a variety of methods based on data processing, including algorithms for filtering, noise reduction, feature extraction and pattern recognition, for example, have been proposed. Juarez et al. [3] improved the SNR by averaging the Rayleigh-backscattering curves. Bao and co-workers [4,5] proposed a moving-average together with moving-differential method, as well as a wavelet denoising method to reduce the average number of nuisance alarms. Spatial response traces, which are generated by sequentially connecting points collected from the same places on the Rayleigh-backscattering curves, reflect changes in the sensing fiber at different positions. By processing the spatial response traces using a power spectrum analysis method, Zhang and co-workers [6] improved the SNR and decreased the nuisance-alarm rate. Zhu et al. [7] were able to distinguish among the three patterns of climbing, kicking and watering by using a multi-feature recognition method. Sun et al. generated an image from the ϕ-OTDR data by taking the position of the sensing fiber as the horizontal axis and time as the vertical axis and representing different levels of optical power on a color scale and applied image processing algorithms, such as those for edge detection and morphological feature extraction, to this image [8,9]. These methods have greatly reduced the nuisance-alarm rate, but are limited by the single information source, which is determined by the fixed pulse-width.

To acquire more abundant and comprehensive information about intrusion events, other methods have been proposed in which ϕ-OTDR has been multiplexed with other distributed intrusion sensors. Rao et al. [10] presented a configuration based on a combination of ϕ-OTDR and polarization-sensitive OTDR, while Liang et al. [11] presented one that multiplexed ϕ-OTDR and a Michelson interferometer. In addition, four different multiplexing structures of ϕ-OTDR and a Mach–Zehnder interferometer have been implemented [12,13,14,15]. However, the configurations of these multiplexing methods are so complicated that to date, multiplexing has been restricted to the case of just two sensors.

By taking account of the fact that the possibility of simultaneous polarization fading on multiple pulse-widths is extremely low, in this paper, we propose a pulse-width multiplexing method that overcomes the quantitative restrictions on multiplexing sensors. The idea behind the proposed method is to inject light pulses with different pulse-widths into the sensing fiber, regard the data acquired at different pulse-widths as the outputs of different sensors and then process these data using a multisensor data-fusion algorithm. The essence of this approach is that a ϕ-OTDR system assisted by pulse-width multiplexing is able to realize the multiplexing of infinite sensors and thus reduce the nuisance-alarm rate to a very low level.

The remainder of this paper is organized as follows. Section 2 describes the principle of the pulse-width multiplexing method. Section 3 establishes an experimental four-pulse-width multiplexing configuration to verify the proposed method. The experimental results and a discussion are presented in Section 4, and Section 5 concludes the paper.

## 2. Principle

**Figure 1:**
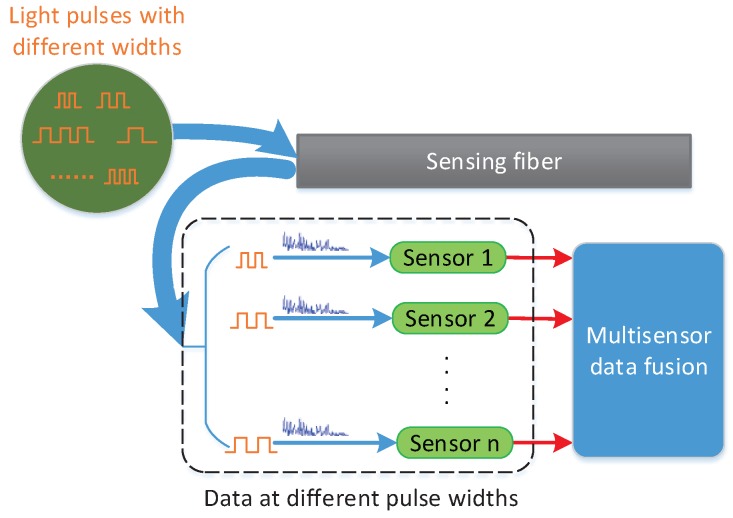
Schematic diagram of the pulse-width multiplexing method.

The principle of the proposed pulse-width multiplexing method is presented schematically in Figure 1. Light pulses of different widths are injected into the sensing fiber and generate Rayleigh-backscattered light, which is detected by the photodetector. While the pulse propagate within the fiber, the Rayleigh-backscattered light varies, and comprehensive information about intrusion events can be acquired. To take full advantage of the differences among data, data acquired at different pulse-widths are regarded as the outputs of different sensors and are then processed by a multisensor data fusion algorithm. The primary aspects of the proposed method, including the influence of pulse-width, the optical structure and the multisensor data fusion algorithm, are discussed in the following.

### 2.1. Influence of the Pulse-Width

The differences among data at different pulse-widths are the basis of the proposed method. The relationships among them can be deduced from a one-dimensional impulse response model of backscattering. Under the assumption that the light pulses are monochromatic and rectangular in shape and ignoring the Rayleigh-backscattered light above second order, the electric field of the optical wave received by the photodetector can be calculated as:

(1) $$E\left(t\right)=\sum_{i=1}^{N}a_{i}\exp\;\left(-\frac{\alpha }{2}\frac{c\tau_{i}}{n}\right)\mathrm{rect}\;\left(\frac{t-\tau_{i}}{T_{p}}\right),$$

where:

(2) $$\mathrm{rect}\left(x\right)=\left\{\begin{array}{cc} 1, & 0\leq x\leq 1,\\ 0, & \mathrm{otherwise}, \end{array}\right.$$

where *N* is the total number of scatterers, *c* is the speed of light in a vacuum, $T_{p}$ is the pulse-width, α and *n* are the attenuation constant and refractive index of the fiber and $a_{i}$ and $\tau_{i}$ are the amplitude and time delay of the *i*-th scattered wave.

The backscattering power of the ϕ-OTDR system, which is proportional to $E^{2}\left(t\right)$, can be divided into two categories: $I_{c}\left(t\right)$ and $I_{v}\left(t\right)$. The difference between $I_{c}\left(t\right)$ and $I_{v}\left(t\right)$ is that $I_{c}\left(t\right)$ represents the complex sum of optical powers from independent scattering centers, while $I_{v}\left(t\right)$ represents the complex sum of interference powers among scattered waves from different scattering centers. Because $I_{c}\left(t\right)$ is insensitive to phase changes induced by intrusion events, we concentrate on $I_{v}\left(t\right)$, which can be calculated as follows [16]:

(3) $$\begin{array}{cc} I_{v}\left(t\right)= & 2\sum_{i=1}^{N}\sum_{j=i+1}^{N}a_{i}a_{j}\exp\;\left[-\frac{\alpha }{2}\frac{c(\tau_{i}+\tau_{j})}{n}\right]\\ \times \cos\varphi_{ij}\mathrm{rect}\;\left(\frac{t-\tau_{i}}{T_{p}}\right)\mathrm{rect}\;\left(\frac{t-\tau_{j}}{T_{p}}\right), \end{array}$$

where $a_{i}$ is the amplitude of the *i*-th scattered wave and $\varphi_{ij}$ is the phase difference between the *i*-th and *j*-th scattered waves. $\varphi_{ij}$ is determined by the distance between the *i*-th and *j*-th scatterers, $l_{i}-l_{j}$, such that $\varphi_{ij}=2\omega n\left(l_{i}-l_{j}\right)/c$, where ω is the optical frequency. Because both the refractive index of the fiber *n* and $l_{i}-l_{j}$ are changed when an intrusion event occurs over the sensing fiber, $\varphi_{ij}$ is sensitive to intrusion events, thereby endowing the ϕ-OTDR system with the ability to detect such events.

As an important parameter of ϕ-OTDR, the width of the launched light pulses also affects the value of $I_{v}\left(t\right)$. If the number of scatterers within the pulse duration is denoted by *M*, then $I_{v}\left(t\right)$ can be rewritten as

(4) $$I_{v}\left(t\right)=2\sum_{i=1}^{M}\sum_{j=i+1}^{M}a_{i}a_{j}\exp\;\left[-\frac{\alpha }{2}\frac{c(\tau_{i}+\tau_{j})}{n}\right]\cos\varphi_{ij}.$$

When the pulse-width is tuned, the value of $I_{v}\left(t\right)$ varies even when the other parameters of ϕ-OTDR, as well as the environment remain completely unchanged, because *M* and $l_{i}-l_{j}$ both change with the pulse-width. In previous research, we have investigated the influence of the pulse-width, both theoretically and experimentally. One of our experimental results shows that the amplitude of $I_{v}\left(t\right)$ gradually increases with pulse-width when vibrations of fixed amplitude and frequency are applied to the sensing fiber. However, when a laser with fixed-frequency drift noise is employed in ϕ-OTDR, the frequency, not the amplitude, of $I_{v}\left(t\right)$ gradually increases with the pulse-width [17,18]. These results indicate that the features of the signal, as well as the variation of noise with pulse-width have significant effects on the details of the information that can be acquired using a pulse-width multiplexing method.

### 2.2. Optical Structure

**Figure 2:**
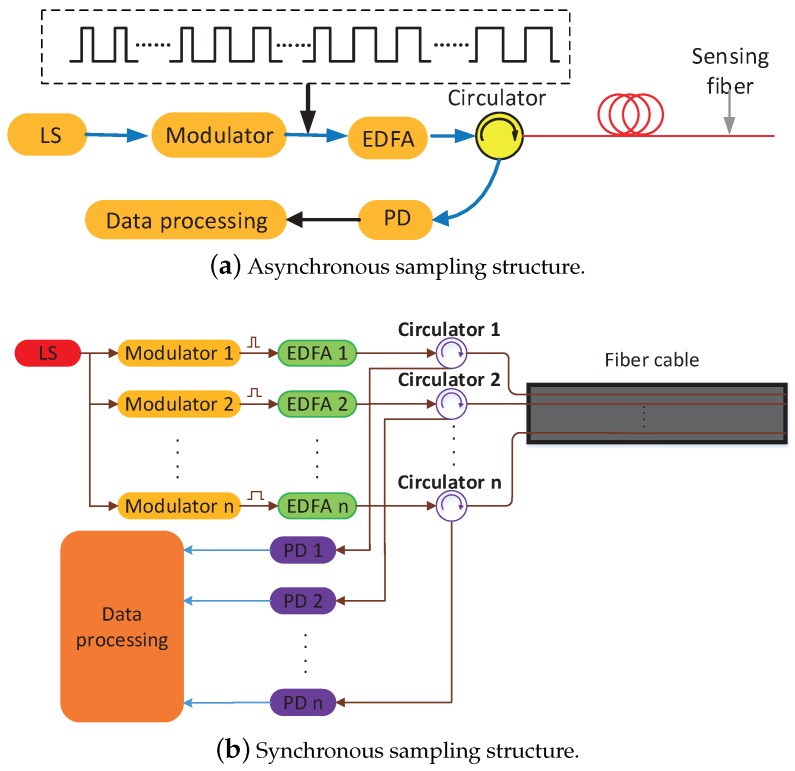
Examples of the optical structure. LS, laser source; EDFA, erbium-doped fiber amplifier; PD, photodetector.

To take full advantage of the pulse-width multiplexing method, the design of the optical structure is critically important. Two examples are shown in Figure 2.

In Figure 2a, a continuous light wave induced by a laser source (LS) is modulated to light pulses, whose widths vary with time. The light pulses are amplified by an erbium-doped fiber amplifier (EDFA) and then injected into the sensing fiber through a circulator. The Rayleigh-backscattered light passes through the circulator again and is then received by a photodetector (PD) and processed by the multisensor data fusion algorithm. This optical structure is an asynchronous sampling structure because, at different pulse-widths, the ϕ-OTDR system collects information on intrusion events at different times.

The other configuration, called a synchronous sampling structure, is shown in Figure 2b. The continuous light wave is divided into *n* parts and is modulated by different modulators to produce light pulses of different widths. These light pulses are amplified by different EDFAs and injected into different fibers in the same cable. When an intrusion event is applied to the sensing cable, all fibers detect the event and guide the Rayleigh-backscattered light to the PDs at the same time. Compared with the asynchronous sampling structure, the synchronous sampling structure requires more devices, such as modulators and EDFAs, as well as PDs, although data processing is easier because the ϕ-OTDR system at different pulse-widths collects information at the same time as the intrusion events. In this paper, an experimental investigation of a synchronous sampling structure is performed to verify the pulse-width multiplexing method.

### 2.3. Multisensor Data Fusion Algorithm

In the pulse-width multiplexing ϕ-OTDR, data at different pulse-widths are regarded as the outputs of different sensors and are processed by a multisensor data fusion technique. This technique has been successfully applied in many fields, such as inertial navigation, multitarget tracking and identification, equipment fault diagnosis and environmental monitoring; thus, much experience has been accumulated on its application, and many algorithms have been developed [19,20,21]. These algorithms can be divided into three categories: data-layer fusion, feature-layer fusion and decision-layer fusion [22]. Data-layer fusion algorithms are able to make full use of the data to provide the most accurate results, but the data processing time is usually long. With feature-level fusion algorithms, which extract the features of the original data and then merge them, there is a loss of data, although these algorithms require less computation time. With decision-layer fusion algorithms, after each sensor makes a decision independently, the data are fused, which greatly reduces the computation time, but gives less reliable results.

In this paper, a feature-layer fusion algorithm based on Dempster–Shafer evidential theory, with the feature chosen to be the SNR, is employed to process the data. The main steps of the algorithm are as follows.

First, the identification framework of the ϕ-OTDR system is set up. This framework consists of four cases: $A_{1}$, intrusion events, alarm; $A_{2}$, intrusion events, no alarm; $A_{3}$, no intrusion events, alarm; $A_{4}$, no intrusion events, no alarm. $A_{1}$ and $A_{4}$ represent correct alarms, while $A_{2}$ and $A_{3}$ represent nuisance alarms and are called a missing alarm and a false alarm, respectively.

Second, basic probabilities are assigned to the elements of the framework. When the ϕ-OTDR system gives out an alarm, the total probability of $A_{1}$ and $A_{3}$ is 100%, such that $m\left(A_{1}\right)+m\left(A_{3}\right)=1$ and $m\left(A_{2}\right)+m\left(A_{4}\right)=0$. $m(A_{1})$ is determined by the SNR and is given by:

(5) $$m(A_{1})=SNR/20+0.5.$$

In this paper, SNR is calculated as 10log(S/N), where *S* and *N* are the maximal and submaximal values of *H*, which is given by:

(6) $$H=\sum_{i=2}^{M}\left|S_{i}-S_{i-1}\right|,$$

where $S_{i}$ is the *i*-th Rayleigh-backscattering curve and *M* is the total number of the curves [18]. When the ϕ-OTDR system does not give out an alarm, the total probability of $A_{1}$ and $A_{3}$ is zero, such that $m\left(A_{1}\right)+m\left(A_{3}\right)=0$ and $m\left(A_{2}\right)+m\left(A_{4}\right)=1$. The value of $m(A_{2})$ is determined experimentally and is usually between 0 and 0.2.

Third, the weight of each element of the framework, ω, can be calculated as:

(7) $$\omega_{i}=\frac{\sum_{j=1}^{n}m_{j}\left(A_{i}\right)}{\sum_{k=1}^{4}\sum_{j=1}^{n}m_{j}\left(A_{k}\right)},$$

where *n* is the number of evidences, or sensors.

Fourth, the similarity between two evidences can be calculated as [23]:

(8) $$\mathrm{sim}\left(m_{i},m_{j}\right)=1-\sum_{k=1}^{4}\omega_{k}\frac{|m_{i}\left(A_{k}\right)-m_{j}\left(A_{k}\right)|}{\max(m_{i}\left(A_{k}\right),m_{j}\left(A_{k}\right))}.$$

Then, the reliability of each evidence is given by:

(9) $$\mathrm{Crd}\left(m_{i}\right)=\frac{\sum_{j=1,j\neq i}^{n}\mathrm{sim}\left(m_{i},m_{j}\right)}{\sum_{k=1}^{n}\sum_{j=1,j\neq k}^{n}\mathrm{sim}\left(m_{k},m_{j}\right)}.$$

Furthermore, from the method of weighted means, the average probability of each element of the framework can be expressed as:

(10) $$m\left(A_{i}\right)=\sum_{j=1}^{n}\mathrm{Crd}\left(m_{j}\right)m_{j}\left(A_{i}\right).$$

The preprocessing of the evidences is now complete.

Finally, the preprocessed data are self-assembled n−1 times according to the Dempster combination rule. The combination formula can be expressed as:

(11) $$m\left(C\right)=\frac{\sum_{A_{i}\cap B_{j}=C}m_{1}\left(A_{i}\right)m_{2}\left(B_{j}\right)}{1-k},$$

where *k* are the conflicting coefficients between evidences and can be calculated as:

(12) $$k=\sum_{A_{i}\cap B_{j}\neq \emptyset }m_{1}\left(A_{i}\right)m_{2}\left(B_{j}\right).$$

The feature-layer fusion algorithm based on Dempster–Shafer evidential theory combines data from multiple sensors and finally gives out the probability of each element in the framework. As a mature and widely-used method, this algorithm has a powerful ability to deal with uncertain information and, thus, to improve the performance of the ϕ-OTDR and enhance the confidence of the system.

## 3. Experimental Setup

### 3.1. Influence of the Pulse-Width

**Figure 3:**
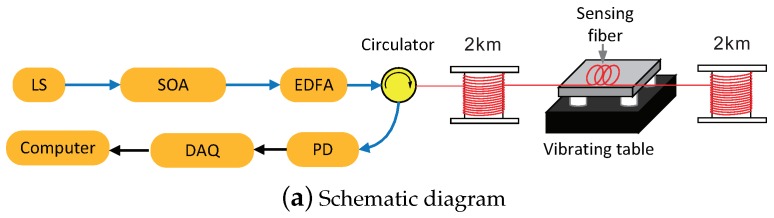

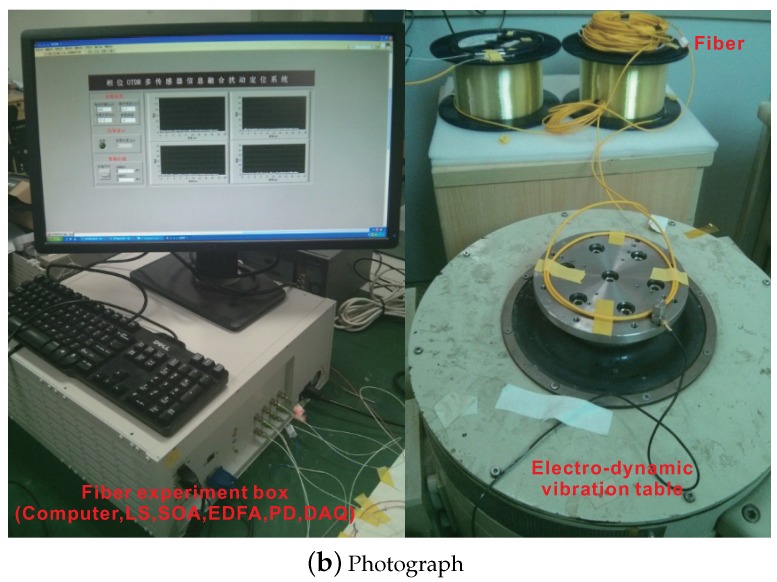
Experimental setup for determining the influence of the pulse-width on SNR. SOA, semiconductor optical amplifier.

Because the differences among data at different pulse-widths are the basis of the proposed method, the influence of pulse-width is tested first in a laboratory setting with the traditional arrangement of the ϕ-OTDR system, as shown in Figure 3. Here, LS is an external cavity laser, with center wavelength 1550.92 nm, linewidth less than 3 kHz and frequency drift less than 10 MHz/min. The continuous light from the laser is converted into light pulses of fixed width by a semiconductor optical amplifier (SOA). These light pulses are amplified by an EDFA and coupled into the sensing fiber via a circulator. The Rayleigh-backscattered light from the sensing fiber is detected by a PD, converted to digital data by a data acquisition (DAQ) card and then processed by a computer. These devices, including the LS, SOA, EDFA, circulator, DAQ card and computer, are integrated in a box to improve the reliability and portability of the ϕ-OTDR system, as shown in Figure 3b. The sensing fiber consists of two thermally-insulated spools of single-mode fiber (both 2 km long) with a 10-m fiber cable spliced between them. The fiber cable is placed on an electrodynamic vibrating table, by the use of which, stable vibrations with specified amplitude and frequency are generated to simulate intrusion events. In the experiment, we document observations of the ϕ-OTDR system when the width of injected light pulses is tuned, and we are thus able to display the influence of the pulse-width.

### 3.2. Four-Pulse-Width Multiplexing ϕ-OTDR System

**Figure 4:**
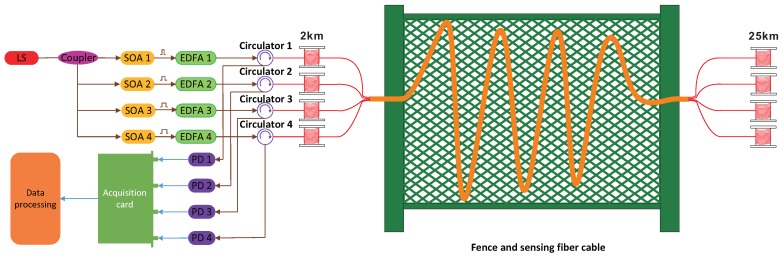
Experimental setup for characterizing the pulse-width multiplexing ϕ-OTDR system.

To verify the effectiveness of the pulse-width multiplexing method, a four-pulse-width multiplexing ϕ-OTDR system with a synchronous sampling structure is set up in the laboratory, as shown in Figure 4. The devices employed in the experiment are the same as in the previous one. Continuous light from the LS is divided into four channels by a coupler and is then modulated into light pulses by SOAs and amplified by EDFAs. The amplified light pulses are injected into different fibers of the sensing fiber, with each fiber consisting of two thermally-insulated spools of single-mode fiber (2 km and 25 km). The total of eight spools of fiber are spliced by a 10 m cable with four optical fibers. The cable is fixed on a wire fence, and intrusion events are simulated by striking this fence. The Rayleigh-backscattered light carrying the information about the intrusion events is detected by PDs, converted into digital data and then sent to the computer. In the experiment, the data from different channels of the sensing fiber are regarded as the outputs of different sensors, to be processed by a multisensor data fusion algorithm based on Dempster–Shafer evidential theory with the aim of minimizing the nuisance-alarm rate.

## 4. Experimental Results and Discussion

### 4.1. Influence of Pulse-Width

**Figure 5:**
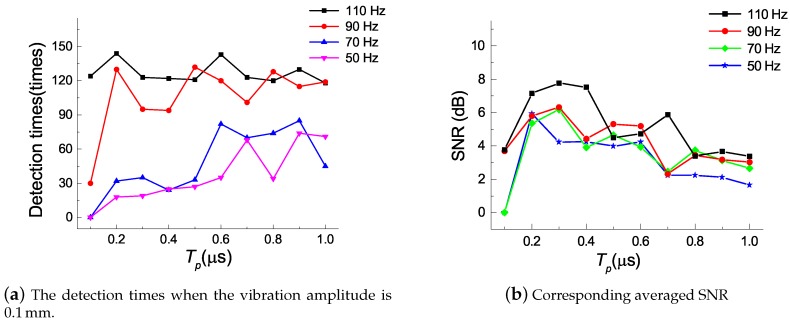
Detection times and corresponding averaged SNR at different pulse-widths.

Detected intrusion events and corresponding averaged SNRs were calculated for a fixed amplitude of vibration of 0.1 mm and frequencies of vibration varying from 50–110 Hz, with the results shown in Figure 5a,b. The acceleration of vibration table, *A*, was determined by the vibration frequency *f* and its amplitude *D*, such that $A={\left(2\pi f\right)}^{2}D$. For the vibration table, the unit of *A* is the acceleration of gravity *g* and 1g=9.8 m/s${}^{2}$, and the unit of Dis usually millimeters. Therefore, the equation can be rewritten as $A\approx f^{2}D/250$. In each case, the electrodynamic vibration table operated for 2 min to simulate intrusion events. As the data processing time of our system was about 0.8 s, the maximum detection time was about 150. The results in the figure show that the missing alarm rate was 100% when only a slight vibration was applied to the sensing fiber, and the pulse-widths were relatively low. However, relatively large pulse-widths were also unable to perfectly detect all intrusion events, because a wide pulse-width may result in a high false alarm rate. Table 1 shows that the false alarm times increased with the pulse-width during 24 h when the vibration table was closed. The influence of the widths of injected light pulses was so complicated that it was difficult to find a single pulse-width that allowed identification of diverse actual intrusion events, which often contain multiple vibrations. Therefore, this paper proposes a pulse-width multiplexing method and demonstrates its capabilities in a laboratory experiment.

### 4.2. Results of the Four-Pulse-Width Multiplexing ϕ-OTDR System

**Figure 6:**
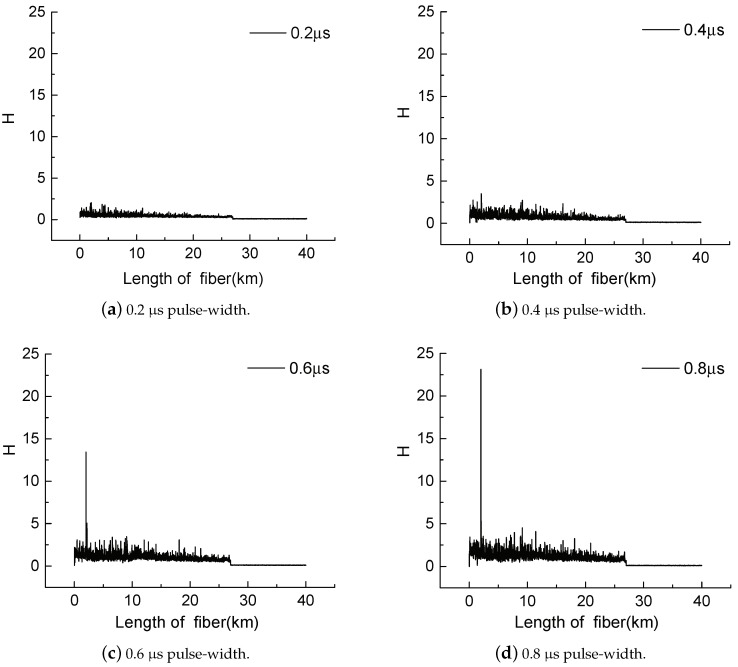
The waveforms of H at different pulse-width.

The pulse-width multiplexing method aids the ϕ-OTDR system by providing more information about intrusion events, thereby minimizing nuisance alarms, including false alarms and missing alarms. In the experiment, the repetition time of light pulses was 400 μs, and the widths were set as 0.2, 0.4, 0.6 and 0.8 μs. The data from different pulse-widths were regarded as the outputs of four different sensors and were then processed by a multisensor data fusion algorithm. According to the algorithm based on Dempster–Shafer evidential theory, as well as experimental experience, if a sensor does not send an alarm, then $m(A_{2})$ and $m(A_{4})$ of the sensor are set as 0.200 and 0.800, respectively. When an intrusion event was simulated by striking the wire fence and a missing alarm occurred, the waveforms of *H*, which is described in Formula (6), under different pulse-widths are shown in Figure 6, and the fusion result is as shown in Table 2a. Table 2b shows the fusion results when no intrusion event was applied to the sensing fiber and a false alarm occurred. These results indicate that the pulse-width multiplexing method is a powerful tool to eliminate false and missing alarms.

The performance of the four-pulse-width multiplexing ϕ-OTDR system was tested in the laboratory and compared with the performance of a traditional ϕ-OTDR system with fixed pulse-width 0.5 μs and that of a four-channel ϕ-OTDR system, with the results shown in Table 3.

The configuration of the four-channel ϕ-OTDR system is the same as in Figure 4, except that the widths of the light pulses injected into the four sensing fibers were all the same and set as 0.5 μs. Each configuration was tested for 72 h to calculate the false-alarm rate, and 500 simulated intrusion events were generated to calculate the missing-alarm rate. Compared with the traditional ϕ-OTDR, the four-pulse-width multiplexing ϕ-OTDR system reduced the nuisance-alarm rate by about 70%. Moreover, a comparison between the experimental results from the four-pulse-width multiplexing ϕ-OTDR system and those from the four-channel ϕ-OTDR system indicates that the ability to reduce the nuisance-alarm rate of the pulse-width multiplexing method is not derived from redundancy.

### 4.3. Discussion

Based on the initial experimental results, the pulse-width multiplexing method may be regarded as a candidate for reducing the nuisance-alarm rate of the ϕ-OTDR system, although there is still scope for further improvement in the performance of the pulse-width multiplexing ϕ-OTDR.

First, in the present approach, the raw data at each pulse-width were not preprocessed by a noise reduction algorithm. If such an algorithm, based, for example, on a moving-average, moving-differential, power spectrum analysis or wavelet denoising method, were used, then the nuisance-alarm rate at each pulse-width would be reduced. In this paper, we have not utilized one of these algorithms, because of the risk of a very low-false alarm rate leading to the need for an excessively long time (maybe several months) to compare the performances of the three configurations in the laboratory.

In addition, the data fusion algorithm employed in this paper is a feature-layer algorithm based on Dempster–Shafer evidential theory, and the feature is simply selected as the SNR. In the process of data fusion, a considerable amount of important information is lost. Therefore, if a data-layer algorithm, which is based on differences between data at different pulse-widths, were used, the nuisance-alarm rate could be further reduced.

Moreover, the multiplexing number of pulse-widths in the experiment is only four, because the synchronous sampling structure requires more devices to establish multiple channels, which would increase the structural complexity and cost. An asynchronous sampling structure would not need additional devices, so an infinite multiplexing number could be realized to further reduce the nuisance-alarm rate. However, the difficulty of data processing increases rapidly in an asynchronous sampling structure, because the data acquired at different pulse-widths reflect information about intrusion events at different times.

## 5. Conclusions

A pulse-width multiplexing method for reducing the nuisance-alarm rate of a ϕ-OTDR system has been investigated. The important aspects of the method, including the influence of pulse-width, the optical structure and the multisensor data fusion algorithm, have been discussed. In laboratory tests with a sensing fiber on a vibrating table, the effects of the pulse-width on the SNR of the ϕ-OTDR data have been observed. By taking the SNR as the feature in a feature-layer algorithm based on Dempster–Shafer evidential theory, a four-pulse-width multiplexing ϕ-OTDR system with a synchronous sampling structure has reduced the nuisance-alarm rate by about 70%. The theoretical analysis and experimental results show that the pulse-width multiplexing method could greatly reduce the incidence of nuisance alarms using a simple configuration and could thus provide a welcome boost to the applications of the ϕ-OTDR system.

## Figures and Tables

**Table 1:** False-alarm times at different pulse-widths during 24 h.

Pulse-Width	100 ns	200 ns	300 ns	400 ns	500 ns	600 ns	700 ns	800 ns	900 ns	1000 ns
False-alarm times	0	0	1	4	3	5	11	8	14	13

**Table 2:** Fusion results.

**(a) Fusion Results with a Missing Alarm**				
**Pulse-Width**	$m(A_{1})$	$m(A_{2})$	$m(A_{3})$	$m(A_{4})$
0.2 μs	0	0.200	0	0.800
0.4 μs	0.544	0	0.456	0
0.6 μs	0.717	0	0.283	0
0.8 μs	0.852	0	0.148	0
**Fusion results**	**0.970**	**0**	**0.030**	**0**
**(b) Fusion Results with a False Alarm**				
**Pulse-Width**	$m(A_{1})$	$m(A_{2})$	$m(A_{3})$	$m(A_{4})$
0.2 μs	0	0.200	0	0.800
0.4 μs	0	0.200	0	0.800
0.6 μs	0	0.200	0	0.800
0.8 μs	0.553	0	0.447	0
**Fusion results**	**0**	**0.004**	**0**	**0.996**

**Table 3:** Experimental results for the nuisance-alarm rate from traditional ϕ-OTDR, four-channel ϕ-OTDR and four-pulse-width ϕ-OTDR.

Optical Structure	Traditional ϕ-OTDR	Four-Channel ϕ-OTDR	Four-Pulse-Width ϕ-OTDR
Knocking times	500	500	500
Detection times	452	473	489
False alarm	7	5	2
Missing-alarm rate	9.6%	5.4%	2.2%
False-alarm rate	2.6 times/km/month	1.9 times/km/month	0.7 times/km/month
